# First case report of a perinatally HIV-infected infant with HIV resistance to dolutegravir associated with tenofovir/lamivudine/dolutegravir use in mothers

**DOI:** 10.1097/QAD.0000000000003653

**Published:** 2023-07-07

**Authors:** Kesner Francois, Joelle Deas Van Onacker, Michael R. Jordan, Ito Journel, Josiane Buteaue, Emmanuel Pierre, Nirva Duval, Georges Dos Santos, Amalia Giron, Harry Geffrard, Omar Sued, Seth C. Inzaule

**Affiliations:** a National AIDS Programme, Ministry of Health, Port-au-Prince, Haiti; b Levey Center for Integrated Management of Antimicrobial Resistance, Tufts University School of Medicine; c Division of Geographic Medicine and Infectious Diseases, Tufts Medical Center, Boston, MA, USA; d National Laboratory Services, Ministry of Health, Port-au-Prince, Haiti; e Service de Virologie, CHU Martinique, Fort-de-France, Martinique; f Independent Consultant; g Pan American Health Organization, Washington DC, USA; h Amsterdam Institute for Global Health and Development, and Department of Global Health, Amsterdam UMC, University of Amsterdam, Amsterdam, The Netherlands.

## Abstract

Perinatally HIV-infected infants can be infected with a drug-resistant virus or select for drug resistance by exposure to sub-therapeutic levels of maternal antiretroviral drugs present in breastmilk or from sub-therapeutic infant prophylaxis. We report a case of dolutegravir resistance detected in a treatment-naive perinatally HIV-infected infant whose mother was receiving tenofovir/lamivudine/dolutegravir. This case was detected during a national survey of HIV drug resistance in Haiti amongst infants testing positive for HIV through the national early infant diagnosis program between April 2020 and March 2021. This unique case underscores the need for prompt management of high viral loads in pregnant and breastfeeding women and supports HIV drug resistance surveillance efforts targeted at antiretroviral therapy-naive infants born to mothers in low-and middle-income countries.

The rapid decrease in plasma levels of HIV RNA in women receiving dolutegravir (DTG)-based antiretroviral therapy (ART), coupled with high levels of viral load suppression, is associated with potential reduction in mother-to-child-transmission and raises hopes for eliminating mother-to-child transmission of HIV [1–5]. However, a small number of perinatally acquired HIV infections has been reported among infants born to mothers receiving DTG-based ART [3]; thus raising concerns for possible integrase inhibitor resistance in infants infected with HIV, despite interventions preventing mother-to-child HIV transmission (PMTCT).

HIV drug resistance (HIVDR) in perinatally HIV-infected infants may occur because of infection with drug-resistant virus, exposure to sub-therapeutic levels of antiretroviral drugs in breastmilk or during infant postnatal prophylaxis (PNP) [6–8]. Prior studies have shown high levels of resistance in ART-naive infants, especially to nonnucleoside reverse transcriptase inhibitors (NNRTIs) used for PNP and to nucleot(s)ides reverse transcriptase inhibitors (NRTIs) [9–11]. However, to the best of our knowledge, there is the first study reporting detectable DTG resistance in an ART-naive infant. The potential for selection of HIVDR in this population has been extrapolated from pharmacokinetic studies showing detectable but sub-therapeutic levels of DTG in breastmilk [12,13]. Moreover, DTG-resistant virus can be selected in mothers with sub-optimal adherence to DTG-based ART and, subsequently, may be transmitted to infants.

Recently, WHO recommended using DTG-containing ART for children aged at least 4 weeks and weighing at least 3 kg [1]; thus, DTG resistance in ART-naive infants may impact treatment outcomes. We report a case of DTG resistance detected in an ART-naive infant identified in Haiti during a national survey of HIVDR in infants less than 18 months of age diagnosed with HIV through the country's early infant diagnosis program.

Following, WHO survey guidance [14], Haiti conducted a survey of HIV drug resistance in infants between April 2020 and March 2021. Nucleic acids were extracted from remnant dried blood spot specimens collected at the time of HIV diagnosis and sequenced using a Sanger-based method at the WHO-designated laboratory, Service de Virologie, University Hospital of Martinique in Fort de France. Sequences were quality-assured following WHO recommendations [15]. HIVDR was predicted using the Stanford HIVdb algorithm Version 9.09. Sequences with low-level, intermediate-level, or high-level resistance were classified as resistant. HIVDR prevalence estimates were assessed using the ‘svy’ utilities in Stata (Stata 12; StataCorp, College Station, Texas, USA) with weighting to adjust for genotypic failure rate.

During the survey period, 5185 infants were born to HIV-infected mothers; 276 (5.3%) of the infants tested positive for HIV. Of these, 143 (52%) had remnant specimens available for HIVDR testing of which 121 (85%) and 105 (73%) yielded a successful quality-assured genotype for protease/reverse transcriptase (PR/RT) and integrase regions, respectively. The mean age of the infants was 8.2 months [95% confidence interval (CI) 7.3–9.2], and the majority (62.7%, 95% CI 54.3–70.3%) had experienced exposure to antiretroviral drugs for PMTCT. Most mothers had received DTG-based ART (70%, 95% CI 58.9–79.5), and most infants (55.2%, 95% CI 46.5–62.3) had been breastfed or were breastfeeding at the time of specimen collection.

The overall prevalence of resistance to EFV/NVP was 50.9% (95% CI 41.7–60.0). The prevalence of any NRTI resistance was 11.2% (95% CI 6.6–18.5) and was driven by ABC and 3TC resistance (10.3%, 95% CI 5.5–17.4), 92% of which was because of the M184V mutation alone which confers low-level resistance to ABC (Fig. 1). No protease inhibitor resistance was detected. The prevalence of resistance to integrase strand transfer inhibitors (INSTI) was 1.1% (95% CI 0.1–7.3) and was due to the R263K mutation detected in one infant (Fig. 1), whose mother was receiving tenofovir/lamivudine/dolutegravir as a fixed-dose combination for the treatment of her HIV infection. R263K causes intermediate-level resistance to DTG, cabotegravir, bictegravir, and elvitegravir and low-level resistance to raltegravir. In addition to INSTI resistance, the infant's virus also had detected resistance to ABC and 3TC because of the M184V mutation and EFV/NVP resistance due to the K103N mutation. This infant was first diagnosed with HIV at 14 months of age and had received ZDV-based PNP. The infant's mother had initiated ART (tenofovir/lamivudine/dolutegravir) 6 months before delivery. Information on the mother's prior ART and prior HIV diagnosis (if different from the one at ART initiation during pregnancy) was unavailable. The mother was generally nonadherent to clinical appointments and had detectable HIV viral load 3 weeks before delivery (7761 copies/ml); no drug resistance test was conducted on the mother's virus. Eight months after delivery, the mother's viral load was less than 1000 copies/ml.

**Fig. 1:**
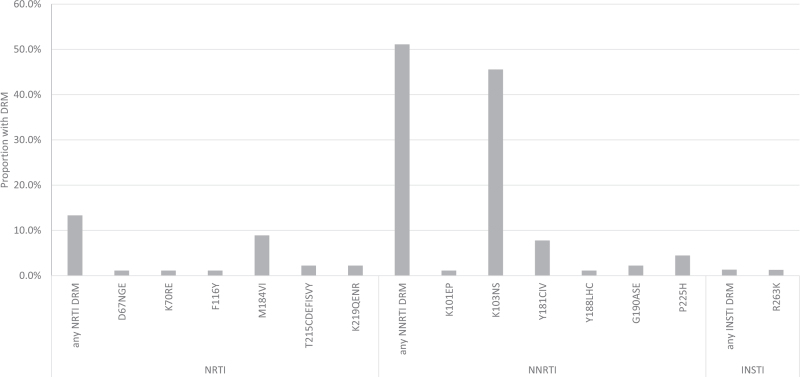
Prevalence and patterns of HIV drug resistance-associated mutations among antiretroviral therapy-naive children less than 18 months of age newly diagnosed with HIV in Haiti.

The prevalence and patterns of HIV drug resistance-associated mutations among infants aged 18 months or less who are newly diagnosed with HIV and treatment-naive in Haiti, 2020–2021 are shown.

In this national survey of HIVDR in ART-naive infants in Haiti, the prevalence of INSTI resistance was very low in this population, occurring in only one of 58 infants whose mothers were receiving DTG-based ART. Nonetheless, the presence of DTG resistance in this one infant merits attention and signals a need for further surveillance in this population as DTG-based ART is rolled out globally because of limited available treatment options and the need for lifelong treatment.

Pharmacokinetic studies have shown that DTG can be passed through the placenta and breastmilk in sub-therapeutic levels raising concerns for selection of drug resistance [12,13]. However, to date, no such case has been reported in literature despite pharmacokinetic studies predicting its possible occurrence [12,13]. In light of these concerns, there is need to strengthen surveillance systems to assess HIVDR in newly infected ART-naive infants to guide optimal treatment and prevention strategies. In the absence of routine drug resistance testing in most settings, close treatment monitoring with timely treatment switch is critical, especially as DTG is scaled up in paediatric populations worldwide. Wherever feasible and affordable, countries may consider prioritizing HIVDR testing in ART-naive, DTG-exposed infants initiating DTG-containing regimens to prevent further selection and accumulation of resistance and to guide optimal regimen selection. Results also underscore the need for ART optimization and prompt management of high viral loads in pregnant and breastfeeding women to further minimize HIV transmission including transmission of drug-resistant virus.

The high prevalence of NRTI and NNRTI resistance is consistent with findings of similar studies [9–11] and underscores the need to fast track adoption of optimal paediatric ART, including regimens using DTG, and close treatment monitoring to ensure successful treatment outcomes in this population [1].

A key limitation of this analysis is the inability to ascertain if the infant was infected with a DTG-resistant virus or if the resistance was selected because of ingesting sub-therapeutic levels of DTG in breastmilk. It is, however, worth noting that, as the infant was not receiving a nonnucleoside reverse transcriptase inhibitor containing PNP, it is likely that the observed resistance may at least be attributed to infection with a resistant virus. Overall, well designed studies are needed to determine the mechanism of DTG resistance in perinatally infected infants and prevention strategies.

In conclusion, we report the first case of detected DTG resistance in an ART-naive HIV-infected infant identified from a nationally representative survey in Haiti. While the near absence of INSTI resistance is highly reassuring, close monitoring of infants initiating ART is required to ensure successful treatment outcomes. Strengthening routine HIVDR surveillance will also help detect any possible increase in the prevalence of DTG resistance in this population and provide information to guide paediatric HIV treatment in countries using a public-health approach.

## Acknowledgements

Authors’ contributions: K.F. was the principal investigator. M.R.J., G.D.S., J.D.V.O., I.J., J.B., E.P., N.D. and H.G., were involved in study design and data collection. M.R.J., A.G., O.S. and S.C.I. conceived the sub-study. S.C.I. performed the statistical analysis. S.C.I., M.R.J. and A.G. wrote the manuscript. All authors provided valuable input to interpretation of the data and critically reviewed the paper for important intellectual content. All authors reviewed and approved the final version of the manuscript.

### Conflicts of interest

There are no conflicts of interest.
